# How to locate the fistula orifice of spinal extradural arachnoid cyst: Surgical experience and clinical outcome

**DOI:** 10.1186/s12893-023-02013-7

**Published:** 2023-06-29

**Authors:** Yu Tian, Yong Chen, Long Chen, Xianghong Meng, Mengmeng Fu, Xin Shi, Yuanxiang Lin

**Affiliations:** 1 Department of Neurosurgery, The First Affiliated Hospital of Fujian Medical University, 20 Chazhong Road, Taijiang District, Fuzhou, 350005 Fujian China; 2 Department of Neurosurgery, Clinical Medical Academy Centre, Shenzhen University General Hospital, Shenzhen University, Shenzhen University, 1098 Xueyuan Avenue, Nanshan District, Shenzhen, 518000 Guangdong China

**Keywords:** Arachnoid cyst, Fistula orifice, Fenestration, Surgical procedure

## Abstract

**Background:**

In clinical practice, spinal extradural arachnoid cysts (SEAC) are relatively rare. The key to the treatment of SEAC is recognize and close the dural defects (fistula orifice), but there is currently no convenient method to locate and identify the fistula. We propose a method for predicting the location of lumbar/thoracolumbar SEAC fistula based on surgical experience, subsequently closing the fistula through posterior unilateral interlaminar fenestration. Evaluating its surgical efficacy and investigated its effect on patient prognosis.

**Methods:**

A stepped approach based on clinical experience is proposed. A retrospective analysis was performed on 6 patients diagnosed with thoracolumbar SEAC disease and treated with posterior unilateral interlaminar fenestration through the position by pre-estimated fistula orifice in our hospital’s Department of Neurosurgery between January 2017 and January 2022.

**Results:**

All patients who received this treatment experienced significantly lower postoperative VAS pain scores and ODI index compared to their corresponding preoperative values (P < 0.01). During the ongoing follow-up after surgery, no unstable vertebral column, adverse effects, or complications were reported.

**Conclusions:**

The use of posterior unilateral interlaminar fenestration for the treatment of large SEAC in the adult lumbar/thoracolumbar segment can reduce spinal cord manipulation and enhance spine stability. The disease can be treated by sealing the fistula orifice through a small fenestra, the position of which is assessed before surgery. This surgical method reduces trauma and improves the prognosis of patients with large SEAC.

## Background

A spinal arachnoid cyst (SAC) is a rare disease that occupies the spinal canal. SAC is typically characterized by increasing paresthesia or radiculopathy [1]. Typically located in the extramedullary region, these cysts can extend into the epidural space via weak regions of the dura [1]. Spinal extradural arachnoid cyst (SEAC) accounts for 1% of primary spinal space-occupying disease [2]. SEAC most often occur in the thoracic spine (65%), followed by the lumbar and lumbosacral spines (13%), the thoracolumbar spine (12%), the sacral spine (6.6%), and the cervical spine (3.3%) [3]. In almost half of the cases, the cysts are situated dorsolateral to the dural sac and extend forth via the intervertebral foramen. Moreover, SEAC are typically associated with diverticular herniation through the dural defect results in the accumulation of cerebrospinal fluid (CSF). However, the specific mechanism by which SEAC develops in the extradural region is unknown. SEAC has been associated with a variety of pathomechanisms. Among them are the congenital splitting of the arachnoid layer, trauma, hemorrhage, inflammation, previous surgery, and lumbar puncture [4, 5].

SEAC symptoms are quite variable. Depending on the location, patients present with neurological compression symptoms [6]. Thoracic SEAC results in radicular pain with a band-like distribution in the chest, whereas lumbar SEAC results in lower back pain with radicular radiation, with some patients experiencing sensory and motor impairments. Sacral SEAC can occasionally result in the anus or urinary incontinence.

While surgery has been recommended for the treatment of symptomatic SEAC, there is no consensus on the best treatment option due to the low incidence of SEAC. According to previous case reports, successful therapy should attempt to seal the cyst’s fistula opening to subarachnoid space (repair of dural defects) [7]. Whether completely removing the cyst wall results in inadequate clinical remission remains debatable [8]. Therefore, it is very important to locate the fistula and perform surgical closure, but there is also the problem of difficulties in locating the dural defects preoperatively [9, 10]. The posterior midline approach frequently results in significant surgical trauma and several complications [11]. We propose a method for predicting the location of thoracolumbar SEAC fistula based on surgical experience, and adults with lumbar/thoracolumbar SEAC were treated successfully with posterior unilateral interlaminar fenestration by pre-estimated fistula orifice. The next sections describe the clinical features, surgical experience, and clinical outcomes.

## Methods

### Patients and study design

A retrospective analysis of clinical data for patients with lower back pain or radicular radiating pain was performed. Patients enrolled in this study if diagnosed with lumbar/thoracolumbar SEAC disease between January 2017 and January 2022 at our hospital’s Department of Neurosurgery. Patients with the following conditions were excluded from the study: (1) evidence of intradural mass lesions or malignant lesions; (2) presence of multiple cystic lesions or metastatic lesions on the cross-sectional MR image; (3) infection, instability, or pain caused by lumbar disc herniation or lumbar spondylolisthesis; (4) cyst size less than two spinal segments. A total of six patients (4 males and 2 females) met the study’s inclusion criteria and were treated with interlaminar fenestration. All surgeries were performed by the same senior surgeon, and each patient provided written informed consent.

### Neuroimaging

All patients underwent preoperative MRI imaging. SEAC was diagnosed if oval-shaped tissue with a long T1-weighted-image (T1WI) and a long T2-weighted-image (T2WI) signal with a clear spinal canal edge was observed. Moreover, the oval-shaped tissue was in sharp contrast to the surrounding adipose tissues, and its signal was identical to that of the CSF. T2-fat-suppression-imaging (T2FS) revealed a hyperintense signal, whereas T2-fluid-attenuated-inversion-recovery (T2FLAIR) revealed a hypointense signal. Due to the difficulty of correctly locating the cyst fistula orifice (the communication point between the cyst and the subarachnoid space) using MRI images, MRI was only used to determine the size of the cyst, and to correctly identify the affected spinal levels.

### Surgical procedure

**Fig. 1:**
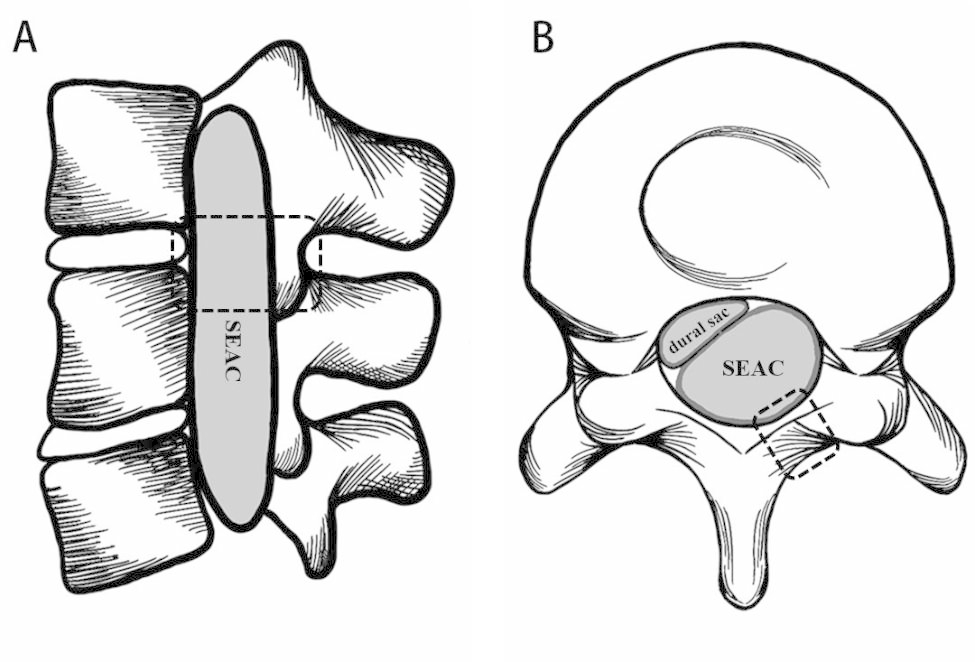
Schematic diagram of the fenestration position. (A & B: We used the mid-upper 1/4 to 2/4 (1/2) of the cyst length shown by MRI sagittal images and the opposite side of the compressed dural sac of the spinal canal by MRI axial images as the estimated location of the fistula orifice. Boxes indicate the position of fenestration.)

The patient was positioned in the prone position, and a stepped approach has been planned. As the first step, the cyst’s size was identified based on its precise location on the affected spinal levels as determined by MRI imaging, and the fistula orifice was estimated based on the surgical experience before the surgery: the cyst was accurately positioned and the mid-upper 1/4 to 2/4 (1/2) of the cyst length was marked as the approximate indicated region of the fistula orifice (Fig. 1). The contralateral side of the compressed dural sac in the indicated region was selected as the vertebral lamina fenestration site during surgery. The unilateral lamina and interspace were exposed by a posterior midline vertical incision. Fenestration was then performed at the correctly indicated level of the spinal lamina. After cutting the ligamentum flavum and scraping off the fatty layers, the complete white thin-walled cyst with a clear visible boundary was discovered. A scalpel was used to fenestrate the cyst wall and drain the colorless transparent liquid. Following that, the cyst wall was peeled from edge to upper and lower sides. The location of the fistula orifice was established. The nerve root was pushed into the fistula orifice (if there was herniation of the nerve root).

**Fig. 2:**
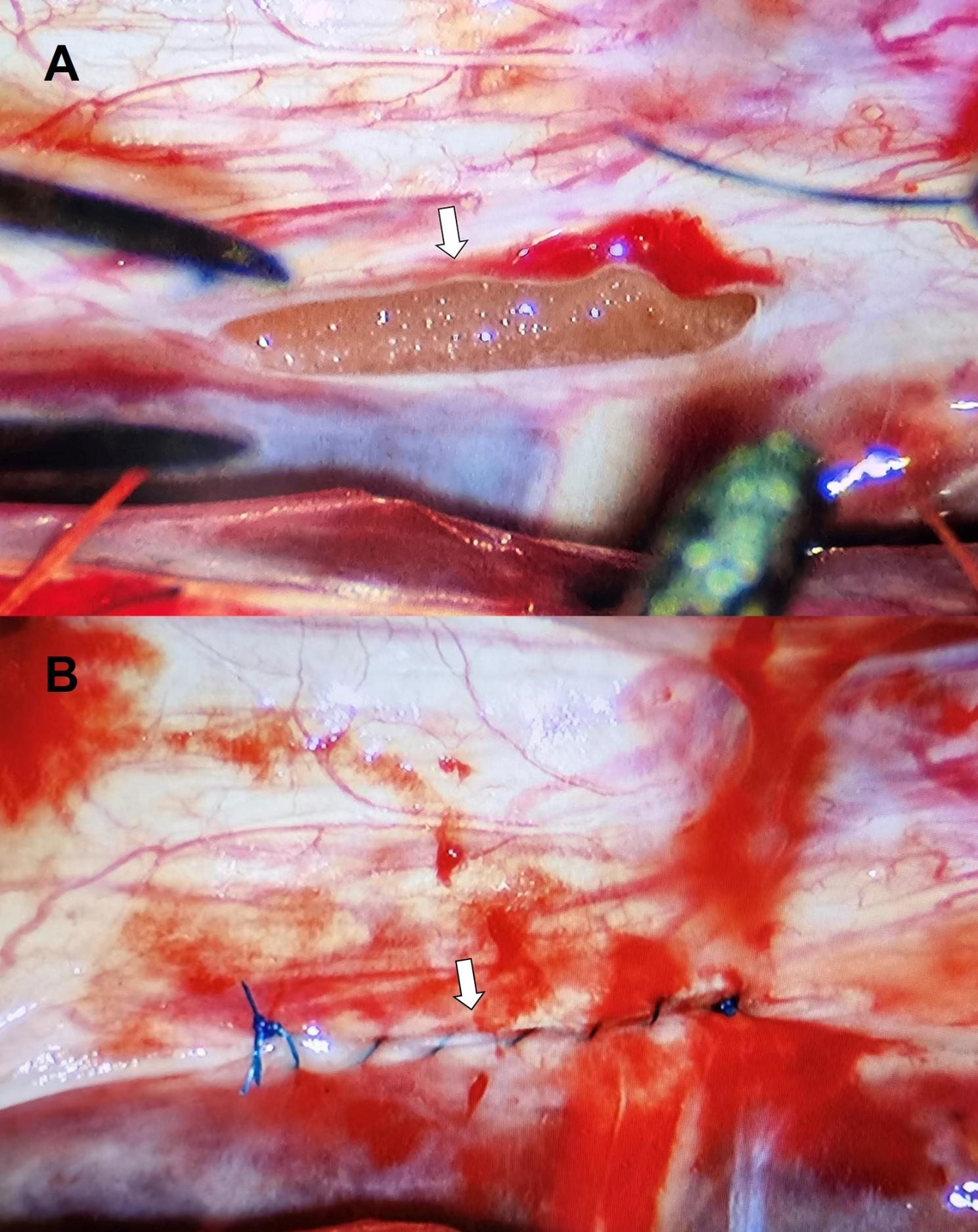
A: The fistula location of the cyst was found after interlaminar fenestration, which was lined with gelatin sponge to avoid damage to the spinal nerve during suturing. (Arrow: the location of fistula orifice.) B: the suturing of the fistula

**Fig. 3:**
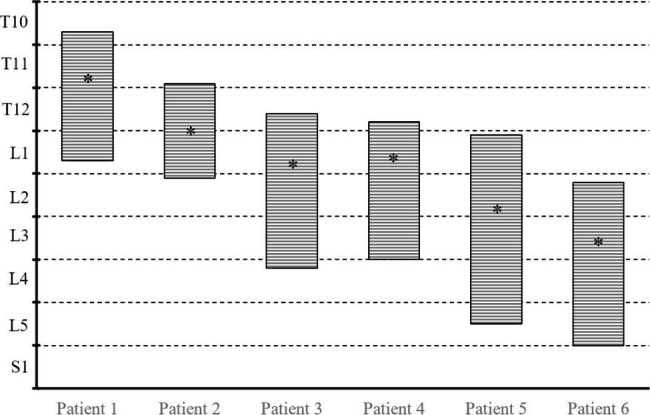
The general spinal segments information of the patient’s cyst. (*: The location of the fistula orifice.)

And if cannot find the fistula orifice by fenestration, second step including extended fenestration or hemilaminectomy/laminectomy and try to find the fistula orifice or total resection of the cyst would be proceeded with. Figure 2A shows the fistula of the SEAC, which was lined with gelatin sponge to avoid damage to the spinal nerve during suturing (Fig. 2A inside the fistula). Primary sutured closure of the orifice and reinforced the fissure with muscles (Fig. 2B). After establishing that there was no CSF leaking from the dural defect, the wound was closed in layers. In all the six patients we included, the fistula orifice was found and closed through the fenestration site (Fig. 3), thus eliminating the need for the second step.

### Post-operative course

After surgery, all patients were examined using MRI imaging to determine the surgical outcome. The visual analogue scale (VAS) was used to assess changes in back and leg pain seven days following surgical decompression for all patients. VAS scores were graded on a scale of 1 to 10, with 1 indicating no pain and 10 indicating the worst pain possible. The Oswestry disability index was used to measure functional improvement (ODI). The ODI index (0 ~ 100%) suggested that those with higher scores had a higher level of disability. The paired-samples t-test was used to examine changes in VAS or ODI parameters before and after surgery. P < 0.05 indicated that the difference was statistically significant (significance level = 0.05). The spine stability was measured (hemilaminectomy/laminectomy) using localized anteroposterior curvatures of > 12° or scoliotic curves of > 15° with or without coronal/sagittal plane translation at the operative segment, which was identified as the unstable vertebral column using CT/X-ray image.

## Results

Six patients had postoperative MRI to confirm the removal of associated cysts. Postoperative pathology showed arachnoid tissue that was definitively identified as an arachnoid cyst (Table 1; Fig. 3).

**Table 1:** Patient characteristics

Patient	Age (years)	Gender	Location	Fenestration Location	Neurological Status	Surgery Outcome
Patient 1	35	Female	Lower T10- Lower L1	Lower T11	Back pain / Progressive paraplegia	Improved
Patient 2	42	Female	Lower T11- Upper L2	T12-L1	Back pain	Complete recovery
Patient 3	45	Male	Lower T12-Upper L4	Lower L1	Back pain / Paraparesis	Improved / Leg numbness
Patient 4	38	Male	Lower T12- Lower L3	Mid L1	Back pain / Paraparesis	Complete recovery
Patient 5	52	Male	Upper L1-Mid L5	Lower L2	Back pain / Leg pain	Complete recovery
Patient 6	54	Male	Upper L2- Lower L5	Mid L3	Leg pain	Improved

**Fig. 4:**
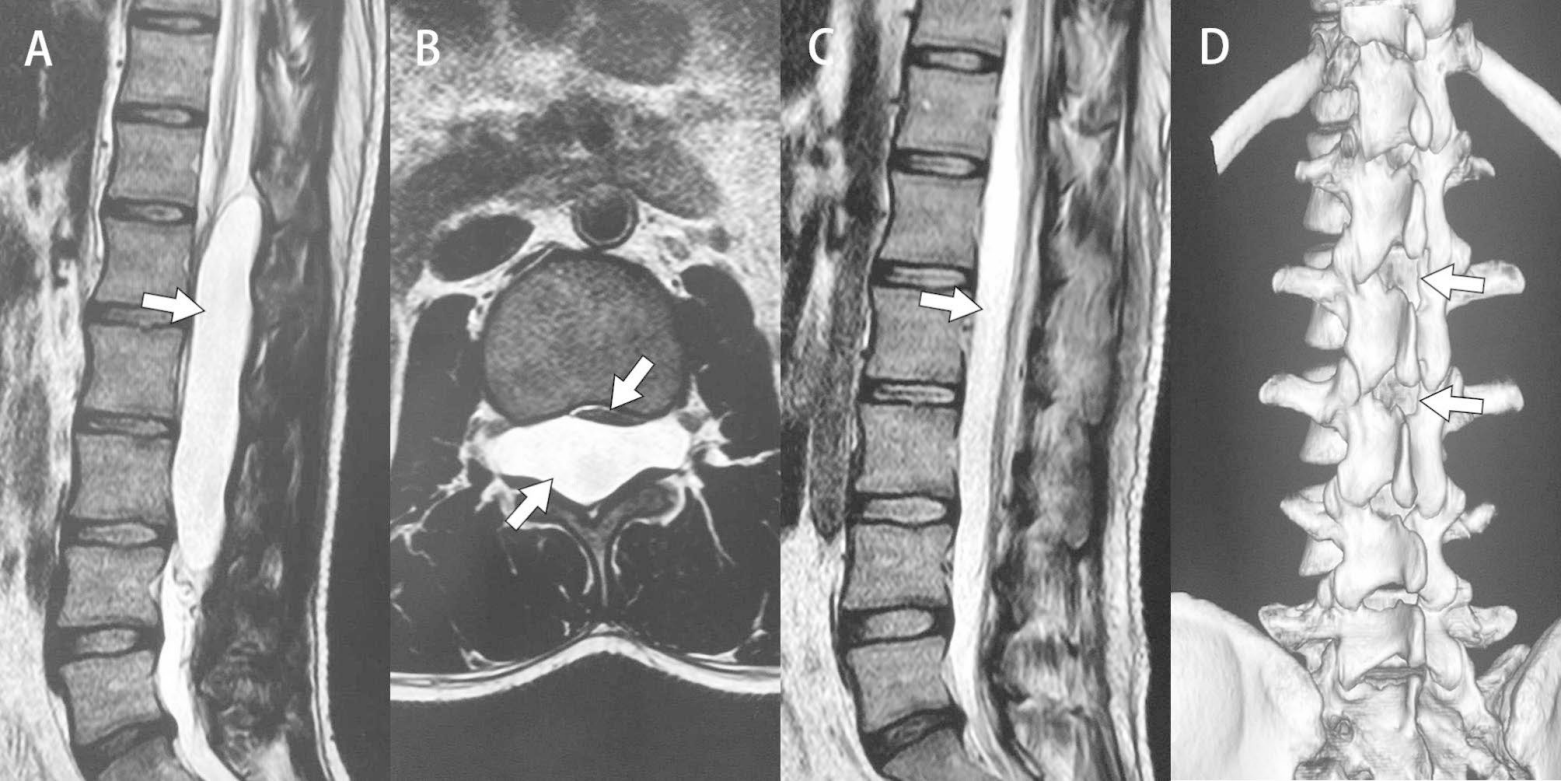
Patient 3 MR imaging data. A: Preoperative sagittal T2WI image. (Arrow: A large SEAC from lower T12 to upper L4.) B: Preoperative axial T2WI image. (Upper arrow: The compressed dural sac. Lower arrow: The SEAC was located dorsally within the spinal canal and severely compressed the cord.) C: Postoperative sagittal T2WI image. (Arrow: The cyst has been completely removed and the space of the spinal canal was also restored.) D: 3D reconstructed image of the spine. (Upper arrow: The position of the lamina fenestration, we closed the fistula orifice and removed the upper part of the cyst wall. Lower arrow: The position of the lamina fenestration, we removed the lower part of the cyst wall.)

Seven days following surgery, five patients reported complete alleviation of lumbar or back pain, complete restoration of muscle strength in their lower limbs, and the ability to walk normally. Lumbar pain was considerably decreased in one patient, with a slight numbness in the outer lower limbs, but muscular strength was completely restored, and the patient was able to live a normal life (Fig. 4). The mean VAS score for back pain reduced significantly postoperatively from (4.50 ± 1.05) to (1.83 ± 0.75) (P<0.01). The mean VAS score for leg pain also decreased from (3.17 ± 0.75) preoperatively to (0.67 ± 0.82) postoperatively (P<0.01). The ODI decreased from (37.67 ± 14.00) % preoperatively to (12.00 ± 9.96) % postoperatively (P < 0.01) and there was no evidence of an unstable vertebral column (Table 2). The minimum follow-up period was 11 months, and the longest was 64 months. The average time of follow-up was 43 months, and the median time was 38 months. At the time of follow-up, the patient was healthy with significantly reduced numbness and no pain. Furthermore, no adverse effect or complication occurred during the continuous follow-up period following surgery. The subsequent patients will be followed for an extended period.

**Table 2:** Clinical outcomes of the patient

	Pre-operative scores	Post-operative scores	*t*		P
Back pain VAS score	4.50 ± 1.05	1.83 ± 0.75		12.65	< 0.001
Leg pain VAS score	3.17 ± 0.75	0.67 ± 0.82		11.18	< 0.001
ODI score (%)	37.67 ± 14.00	12.00 ± 9.96		13.57	< 0.001

## Discussion

SEAC is rare and has been rarely reported in the literature. Therefore, SEAC has been referred to in the previous literature by a variety of names, including arachnoid diverticula, leptomeningeal cysts, pseudomeningocele, meningoceles, diverticula, localized adhesive arachnoiditis, and epidural cyst. Currently, there is a consensus to use the term ‘Spinal arachnoid cyst (SEAC)’ to describe the disease [12]. SEAC can be classified into three types, according to Nabors et al.: (1) extradural meningeal cysts without spinal nerve root fibers; (2) extradural meningeal cysts with spinal nerve root fibers; and (3) spinal intradural meningeal cysts [13]. These cysts are most frequently found in the intradural extramedullary region, with cysts in the extradural region being an uncommon occurrence [1]. The majority of SEACs are found in the thoracolumbar region of the spine, and they are more prevalent in male patients. The neurological examination revealed mostly back or leg pain, as well as a pattern of spastic or flaccid paraparesis and paraplegia. The majority of individuals with SEAC experienced symptoms that persisted for many months. Clinical symptoms fluctuated in one-third of the patients, and only a small number of individuals obtained long-term remission from the disease. Cyst obstruction and recanalization may be responsible for the variable alleviation and return of symptoms. Because its clinical symptoms are variable, nonspecific, and sometimes the disease progresses more slowly, it is frequently misdiagnosed or delayed. However, because of its low prevalence and the limited number of clinical reports, the clinical mechanism of its occurrence remains unclear. According to the findings of existing literature, the protrusion of non-traumatic SACs form the structurally weak part of the dura mater is a congenital condition [14]. The most widely recognized mechanism, on the other hand, is an enlargement of the cyst, which is mainly caused by the ball-valve mechanism. A fistula orifice on the cyst protrudes from the weak part of the dura mater, causing the cyst to bulge out. When the patient coughs hard and abdominal pressure increases, CSF flows into the epidural arachnoid sac from the fistula orifice of the dural defect [14, 15]. Studies have shown that a fistula orifice is present in virtually all instances of SACs. Fistula orifices can be divided into 2 types [13] : those that function like valves and those that do not function like valves. The presence of a valve-like fistula orifice generally results in clinical symptoms of SACs because the CSF in the cyst is unable to flow back into the subarachnoid space. Because CSF may flow freely via a non-valve-like fistula orifice and the pressure in the cyst is the same as that in the dural sac, symptoms of SACs are unlikely to occur. In addition to the presence of a unidirectional valve mechanism [15, 16], other possible etiologies have been proposed in the current literature, including (1) secretions of the cells present in the cyst wall [17, 18]; (2) pathologic distribution of arachnoid trabeculae leading to the formation of a diverticulum [19].

The use of imaging examination has been successful in diagnosing SEAC. Clinical data indicates that MRI is the preferred diagnostic modality. The cystic content had a signal intensity that was similar to that of the CSF and was characterized as hypointense on the T2FLAIR image and hyperintense on the T2FS image. Following the injection of a contrast agent into the subarachnoid space, Magnetic Resonance Myelography (MRM) and CT Myelography (CTM) may be used to determine the morphology of the cyst. The compression of the spinal cord and subarachnoid space can be seen using these techniques. MRM, on the other hand, is more invasive than MRI, which is why it is not routinely used. In most cases, plain X-ray radiographs can only be used to detect lesions in the spinal canal that are occupying space and growing slowly. Therefore, the diagnostic specificity of the disease determined by X-ray is poor.

The optimal treatment of SEAC has not yet been established due to its low incidence. There is a consensus that surgical treatment for symptomatic SEAC can be beneficial. It has been hypothesized that defining and sealing the fistula orifice between the cyst and the subarachnoid space is an effective treatment strategy [9]. Regarding advanced fistula orifice localization, the methods currently used are mainly imaging examination techniques, such as MRI and MRM. Current research, on the other hand, indicates that techniques have certain limitations. The sensitivity for determining the location of the fistula orifice is low for both MRI and MRM [9, 10]. Numerous reports have failed to locate the fistula opening using MRI and MRM. As a result, the location of the fistula orifice determined with limited sensitivity and specificity using an MRI scan [20]. Because MRM is an invasive examination procedure, patients are often exposed to certain examination risks. It appears to be difficult to estimate the time range within which the cyst develops. Previous studies reveal that scanning immediately after the injection of contrast agent only exposes half of the SEAC, but delayed scanning reveals virtually the entire cyst [21]. False-negative results can be obtained when the valve-like fistula orifice is too narrow for the contrast agent to pass through. With regards to the improvement of fistula orifice positioning technology, Neo et al. [22] discovered pulsing flow voiding by thorough monitoring of T2-weighted images using the cinematic magnetic resonance imaging (cine-MRI) technique before surgery. Researchers have discovered that while cine-MRI cannot always identify the communication site, myelography and CTM do have the ability to detect the communication site in some instances [12, 23]. And if the real-time leak site is not clearly observed, detection by the stepped approach we describe is recommended. Moses et al. [24] used 3D rotational fluoroscopic guidance to puncture the SAC in the case of injecting intracystic contrast under real-time fluoroscopy to determine the location of the fistula orifice. The use of intraoperative endoscopy proved to be beneficial as well [23]. Furthermore, in certain cases, intraoperative full-endoscopic uniportal technique [25] or camera-equipped epiduroscope neural laser decompression [26] were used during the surgery.

From the above analysis, there is no single technique for the precise localization of fistula orifices in SEAC, and each method had its own set of shortcomings. Based on our previous surgical experience and literature review, we propose a stepped approach for SEAC: MRI was routinely performed before the patients underwent surgery. If a fistula orifice failed to be directly displayed on the image, the position of the orifice was estimated. The location of the fistula orifice was estimated to be in the mid-upper 1/4 to 2/4 (1/2) of the cyst length as indicated by MRI sagittal images and on the opposite side of the compressed dural sac of the spinal canal as demonstrated by MRI axial images (Fig. 1). Therefore, SEAC can be treated with fenestration by close the fistula at the estimated location. If no fistula was found, a second step of extended fenestration or hemilaminectomy/laminectomy was performed for treatment, and additional levels would need to be explored most likely below or above the initial laminotomy and often the fistula can be detected. In all the cases we included, fistula was identified and closed at the predicted site, so the second step was not performed.

**Fig. 5:**
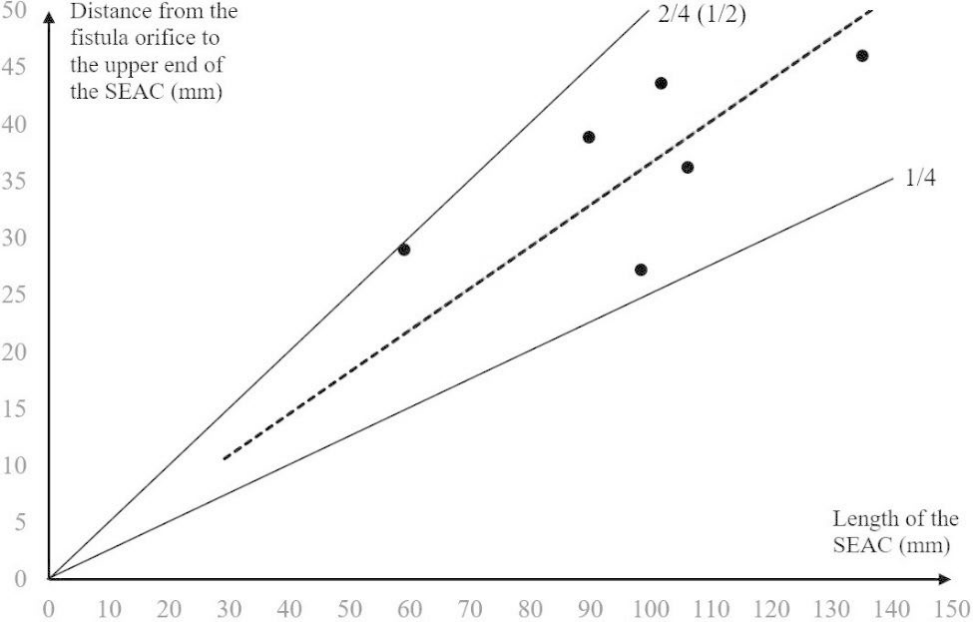
The relationship between the SEAC length and the location of the fistula orifice

During clinical practice, the majority of SEAC patients had fistula orifices in the middle and upper part of the cyst. According to our surgical experience, most of the fistula orifices were restricted to the mid-upper 1/4 to 2/4 (1/2) of the cyst length and may be identified by opening the estimated location on the vertebral lamina (Fig. 5). Table 3 summarizes the length of the cyst and the location of the fistula orifice based on existing literature on single SEAC with lengths larger than two segments (Part of the literature only describe the general segment without clearly describing the location of the fistula orifice and specific length of the cyst). Although SEAC is rare, we discovered that the majority of the fistula orifices were located in the middle and mid-upper part of the cyst based on existing case reports. In addition, the fistula of SEAC across the thoracolumbar segment are often located at T12 to L1 (Table 3). The explanation for this is still unknown, although we speculated that it may be caused by the gravity of the CSF and the pressure generated in the epidural space when the CSF leaks from the weak parts of the dura in the human body with upright walking posture. When the cyst first bursts through the dural defect, it generally takes on a smaller spherical form. Because of the increased abdominal pressure, a large amount of CSF will flow into the cavity, where the valve-like fistula orifice causes leakage of the CSF. As the valve shuts when the abdominal pressure decreases, the cystic CSF remains in the cavity. This mechanism causes the cyst cavity to progressively expand. Because of the epidural space constraint, the cyst will continue to develop in a long oval form above and below the fistula opening. The lower half of the cyst is longer than the upper half because of the patient’s upright walking style and the gravity of the CSF in the cyst. Therefore, according to our observations and analysis, the fistula orifice may be located around the mid-upper 1/4 to 2/4 (1/2) of the cyst length. Furthermore, during the procedure, the fistula orifice was frequently discovered around a nerve root sleeve. This indicates that the cyst is more prone to form where the dura is weak. This phenomenon can precisely locate the fistula orifice.

**Table 3:** The length of the cyst and the location of the fistula orifice reported in the literature (lengths larger than two segments)

Author (Published Year)	Age (years)	Gender	Length	Fistula orifice Location	Neurological Status	Surgery Outcome
Neo, M. et al. [22] (2004)	29	Female	T11-L3	L1	Right lower leg weakness	Complete recovery
Miyamoto, M. et al. [32] (2006)	50	Male	T11-L1	T12	Paraparesis and numbness	Sensory disturbance improved
Tureyen, K. et al. [6] (2009)	25	Male	T11-L2	T12	Progressive back pain radiating to both legs	No sensory and motor deficit
Chio, S. W. et al. [29] (2013)	59	Male	T12-L3	L1	Leg radiating pain and paresthesia	Symptoms gradually subsided
Chio, S. W. et al. [29] (2013)	51	Female	T11-L2	T11-T12	Left buttock pain and paresthesia	Symptoms relieved gradually
Woo, J. B. et al. [11] (2016)	72	Female	T12-L4	L1	Low back pain and leg radiating pain	Symptoms gradually subsided
Woo, J. B. et al. [11] (2016)	33	Female	T12-L2	L1	low back pain and right leg radiating pain	Complete recovery
Lim, M. S. et al. [28] (2016)	43	Male	T10-L1	T11	Persistent back pain/ Lower limb parasthesia	Recurrence after 6 months
LEE, Y. W. et al. [33] (2018)	51	Male	T7-T9	T8	Bilateral lower limbs hypermyotonia	Recurrence after 2 weeks
Ying, G. Y. et al. [10] (2019)	26	Male	T10-L3	T12	Right lower limb weakness	Improved
Kojimahara, Y. et al. [34] (2019)	49	Female	T11-L1	T12	Hip and buttock pain	Complete recovery
Oyemolade, T. A. et al. [35] (2019)	12	Female	T4-T8	T7	Lower limb weakness associated spasms and paraesthesia	Recovery
Özdemir, M. et al. [36] (2019)	22	Male	C7-T3	T2	Progressive neck pain	Finger muscle strength had returned to normal
Lee, S. et al. [7] (2020)	25	Male	T11-L3	T12	Impotence	Symptoms improved
Santipas, B. et al. [37] (2020)	10	Male	T1-L4	T4	Lower limbs weakness and numbnes	Complete recovery
Chaturvedi, J. et al. [38] (2020)	37	Male	T11-L2	T12-L1	Lower extremity paraparesis	Completely asymptomatic.
Zhang, P. et al. [39] (2022)	11	Female	T10-L3	T12	Lower limbs weakness	Complete recovery
Savage, A. J. et al. [40] (2022)	40	Female	T6-8	T7	Back and leg pain	Significant improvements
Ouyang, T. et al. [41] (2022)	52	Male	T11-L2	L1	Left lower extremity weakness	Muscle strength returned to normal
Ouyang, T. et al. [41] (2022)	17	Female	T11-L2	T12	Lower back pain	Muscle strength returned to normal
Ouyang, T. et al. [41] (2022)	41	Male	T11-L2	T12-L1	Lower extremities numbness and sexual dysfunction	Symptoms improved
Ouyang, T. et al. [41] (2022)	34	Male	T11-L2	T12	Lower left limb pain and numbness	Recovery

(Most of the literature only describe the general segment without clearly describing the location of the fistula orifice and specific length of the cyst)

Currently, the most common surgical approach for symptomatic SEAC is the complete removal of the cyst with multi-level laminectomy and repair of any possible dural defects. This invasive surgical technique is associated with a significant risk of kyphotic deformity, particularly in younger patients with large cysts spanning many vertebral levels, which usually results in spinal instability. Although some research suggests that simultaneous spinal fusion might reduce this risk, the operative trauma associated with such procedures is still significant [27, 28]. Although Chio et al. [29] and Woo et al. [11] reported on selective laminectomy for the treatment of SEAC, there is also the problem of difficulties in locating the fistula. MRI imaging was used to locate the cyst on the body surface preoperatively and accurately estimate the location of the fistula orifice. Unilateral fenestration with a small incision and a small fenestra was then performed to access the cyst area to drain the cyst fluid, suture the fistula orifice, and peel away to remove most of the cyst wall. The cyst wall can be sutured with folding in double layers when the fistula orifice is near the defect of the nerve root sleeve. To prevent cyst re-formation, if the cyst wall is particularly weak and CSF still leaks out after reinforcing suture, it can be strengthened with a combination of fibrin sealants glue and muscle or adipose tissue. The location of the fistula orifice was estimated before the operation. Unilateral fenestration was performed using a small fenestra to access the cyst area, suture the fistula orifice, and cut the cyst wall. The cyst was excised while also avoiding its recurrence and preserving the spine’s stability. This complies with the principles of minimally invasive surgical procedures.

The fistula orifice was sutured and most of the cyst wall was peeled away to completely decompress the spinal canal space. However, there is no conclusive evidence on whether the cyst wall should be treated. Most scholars recommend resecting the arachnoid cyst wall as much as possible after fully suturing the fistula orifice. This is because some scholars have found that the disease can be treated by removing the entire cyst wall without locating the fistula orifice intraoperatively [30]. Funao et al. [23] on the other hand, discovered no significant difference in postoperative neurological function recovery between the two methods. There was complete resection of the cyst and closure of the dural defect without cyst resection, however, the latter caused greater damage to the spinal column’s stability due to variations in kyphotic angles. According to the literature, the recurrence of the SEAC is related to the repair of the dural defect but not to the completeness of cyst wall excision [31]. All of this makes us cautious about treating the cyst wall; to reduce the invasiveness of the surgical procedures, the majority of the cyst wall inside the boundaries of the opened fenestra should be eliminated. Because the lumbar epidural has a large space and the adhesion between the cyst wall and surrounding tissues is weak, we propose removing the majority of the cyst wall and closing the fistula orifice to restore the original structure of the epidural space.

## Limitations

Although the proposed surgical technique is less invasive and safe, it does have some limitations. First, the location of the fistula orifice was determined using our extensive surgical experience because fistula orifice sites are not well defined due to a lack of large data and samples to allow extensive research. Second, this method can only estimate the position of the fistula orifice in large (more than two spinal segments) SEAC. Smaller cysts can be located directly without using this predictive fistula orifice technique for direct fenestration. The proposed imaging technology is less sensitive to detecting the fistula orifice. This procedure will be useful in predicting the location of the fistula orifice for the treatment of this disease, however, with the advancement of imaging technology, neuroendoscopy (flexible neural endoscopy) interventional treatment can be used to close the dural defect on the premise of accurate development of the fistula orifice, thus play an important role in the minimally invasive treatment of this disease. This is because the lumbar/thoracolumbar spinal canal space is larger than that of the thoracic and cervical segments. Furthermore, because there are fewer nerves, it is advantageous to undertake small vertebral lamina fenestration and minimally invasive cyst stripping operations, which causes less trauma to the spinal cord. This surgical method was exclusively used on the lumbar section for safety reasons. When researchers gain more surgical experience, they will be able to broaden the treatment to include thoracic and cervical segments. The dorsolateral side of the spinal canal is exposed during this surgery, but due to the limitation of the medial spinous process and the lateral articular process, the operator usually has a small operating space. This surgical technique requires the operator to adapt to the delicate operation of microsurgery in a small area. Therefore, beginners have to face a steep learning curve.

## Conclusions

Currently, there is no standard treatment for SEAC due to its low incidence. The classical approach of fully excising the cyst with multi-level laminectomy destabilizes the spine and increases the risk of postoperative complications. The most critical aspect of the surgery is locating and closing the fistula orifice between the cyst and the subarachnoid space. Therefore, to avoid recurrence of SEAC, treatments should not only decompress the spinal canal by removing the cyst but also shut the fistula orifice. In the present cases, unilateral vertebral lamina fenestration was used to treat adults with large SEAC in the lumbar/thoracolumbar segment. This technique minimized manipulation of the spinal cord by the cyst and improve spine stability. The disease was effectively treated by closing the fistula orifice.

## Data Availability

The data that support the findings of this study are available from the corresponding author upon reasonable request.

## Abbreviations

SAC: spinal arachnoid cyst
SEAC: spinal extradural arachnoid cyst
CSF: cerebrospinal fluid
MRI: magnetic resonance imaging
T1WI: T1-weighted-image
T2WI: T2-weighted-image
T2FS: T2-fat-suppression-image
T2FLAIR: T2-fluid-attenuated-inversion-recovery
VAS: visual analogue scale
ODI: Oswestry disability index
MRM: magnetic resonance myelography
CTM: CT myelography
cine-MRI: cinematic magnetic resonance imaging
